# cDNA Sequence and Fab Crystal Structure of HL4E10, a Hamster IgG Lambda Light Chain Antibody Stimulatory for γδ T Cells

**DOI:** 10.1371/journal.pone.0019828

**Published:** 2011-05-24

**Authors:** Petra Verdino, Deborah A. Witherden, Katie Podshivalova, Stephanie E. Rieder, Wendy L. Havran, Ian A. Wilson

**Affiliations:** 1 Department of Molecular Biology, The Scripps Research Institute, La Jolla, California, United States of America; 2 Department of Immunology and Microbial Science, The Scripps Research Institute, La Jolla, California, United States of America; 3 Skaggs Institute for Chemical Biology, The Scripps Research Institute, La Jolla, California, United States of America; Aston University, United Kingdom

## Abstract

Hamsters are widely used to generate monoclonal antibodies against mouse, rat, and human antigens, but sequence and structural information for hamster immunoglobulins is sparse. To our knowledge, only three hamster IgG sequences have been published, all of which use kappa light chains, and no three-dimensional structure of a hamster antibody has been reported. We generated antibody HL4E10 as a probe to identify novel costimulatory molecules on the surface of γδ T cells which lack the traditional αβ T cell co-receptors CD4, CD8, and the costimulatory molecule CD28. HL4E10 binding to γδ T cell, surface-expressed, Junctional Adhesion Molecule-Like (JAML) protein leads to potent costimulation via activation of MAP kinase pathways and cytokine production, resulting in cell proliferation. The cDNA sequence of HL4E10 is the first example of a hamster lambda light chain and only the second known complete hamster heavy chain sequence. The crystal structure of the HL4E10 Fab at 2.95 Å resolution reveals a rigid combining site with pockets faceted by solvent-exposed tyrosine residues, which are structurally optimized for JAML binding. The characterization of HL4E10 thus comprises a valuable addition to the spartan database of hamster immunoglobulin genes and structures. As the HL4E10 antibody is uniquely costimulatory for γδ T cells, humanized versions thereof may be of clinical relevance in treating γδ T cell dysfunction-associated diseases, such as chronic non-healing wounds and cancer.

## Introduction

T cells of the γδ lineage constitute an enigmatic cell population which links adaptive and innate immunity [1]. Like αβ T cells and B cells, γδ T cells undergo V(D)J rearrangements, but their γδ T cell receptor (TCR) diversity is created to a lesser extent by V gene usage, than by skewing combination events in the CDR3 junctions [2]. Interestingly, some γδ T cell populations have highly restricted V gene usage, preferred pairing of TCR chains, and entirely lack junctional diversity, resulting in the expression of canonical TCRs [3]. γδ T cells have not been shown to recognize peptide/MHC (major histocompatibility complex) complexes or utilize known antigen processing and presentation pathways as for antigen recognition by αβ T cells. Instead, the specialized antigens and antigen recognition requirements for γδ T cells provide unique immunoregulatory and immunoprotective functions [4], [5], [6], [7]. γδ T cells comprise 1–10% of the T cell population in the body; however, in selected tissues they are the majority or only T cell population [8], [9], [10], [11], [12]. Functionally, γδ T cells are believed to perform immune system regulation and surveillance roles, such as tumor cell recognition, maintenance of tissue homeostasis, and tissue repair [1], [9], [10], [12], [13] and act as the first line of defense against infection [7], [14].

Dendritic epidermal γδ T cells (DETC) are the only resident T cell population in the skin [8], [10]. DETC are CD4 and CD8 double negative and do not express the costimulatory molecule CD28 [9], [15]. In fact, the majority of γδ T cell populations do not express CD4, CD8, or CD28 [8], [14], [16] and the absence of these molecules represents an important finding because co-receptors and costimulatory receptors are essential for tuning αβ T cell responses. Several diseases are correlated to dysfunction of costimulation: autoimmune diseases [17], [18], [19], [20], [21], fatal dilated cardiomyopathy [22], lymphoproliferative disorders and multi-organ tissue destruction [23], [24], and common variable immunodeficiency [25].

We generated monoclonal hamster antibodies against proteins expressed on epithelial γδ T cells to identify novel costimulatory molecules that compensate for the lack of traditional co-receptors. Binding of one of those antibodies, HL4E10, to epithelial γδ T cell surface-expressed JAML (Junctional Adhesion Molecule-Like) receptor leads to potent costimulation via phosphoinsositide-3-kinase recruitment, activation of Akt and MAP kinase pathways, and cytokine production, ultimately resulting in epithelial γδ T cell proliferation [15], [26], [27].

Hamsters are widely used to generate monoclonal antibodies because they are less evolutionary related to mouse and rat than these are to each other. Therefore, hamsters can generate good immune responses to mouse and rat antigens, but still yield stable hybridomas after fusion with mouse myeloma cells [28]. However, sequence information for hamster immunoglobulins (Igs) is sparse. To date, only one partial hamster IgM heavy chain DNA sequence [29] and three hamster IgG DNA sequences, that code only for kappa light chains, have been deposited in Genbank: the *Cricetulus migratorius* antibody clones 1F4/3A5-1/4A6 (1F4 light chain: Genbank accession no. S80615, 3A5-1 heavy chain: S80616) [30], 145.2c11 (light chain: U17870, heavy chain: U17871) [31], and H28.710 (light chain: U17165, heavy chain: U17166) [32], [33].

Here, we report the first crystal structure of a hamster IgG Fab fragment and the complete cDNA sequence of the stimulatory antibody HL4E10 which contains the first example of a hamster lambda light chain.

## Materials and Methods

### N-terminal protein sequencing of the HL4E10 hamster IgG

Hybridoma secreting HL4E10 IgG monoclonal antibodies (mAb) were produced by fusing mouse myeloma cells with spleen cells from an Armenian hamster (*Cricetulus migratorius*) immunized with dendritic epidermal T cells (DETC, cell line 7–17), as described elsewhere [15].

N-terminal, amino-acid sequences were obtained from purified HL4E10 IgG (see next section) by Edman degradation (University of Texas Medical Branch, Galveston, USA) of the HL4E10 light and heavy chains. The SDS-PAGE-separated, PVDF-membrane blotted HL4E10 light chain yielded the sequence SYTLTNPPL. The N-terminus of the HL4E10 heavy chain was blocked by pyroglutamate which had to be enzymatically removed prior to Edman degradation. Since the HL4E10 heavy chain was unstable in the standard reducing conditions (i.e. 50 mM Na-phosphate pH 7.0, 10 mM DTT, 1 mM EDTA, 40–75°C for the several hours [34], [35], [36] required for *Pfu* pyroglutamate aminopeptidase (PGAP) activity), we investigated different reducing agents and conditions and found that 50–90% of the HL4E10 heavy chain remains intact after incubation at 40°C for 10 h in the presence of 1–2 mM β-mercaptoethanol.

PGAP deblocking of the HL4E10 heavy chain was achieved as follows: 10 mU lyophilized *Pfu* PGAP (Takara Bio Inc., Japan) was reconstituted in 50 µl of 50 mM Na-phosphate, 5 mM EDTA, 2.5 mM β-mercaptoethanol, pH 7.0 and heat activated by incubation at 55°C for 2 min (heat activation has shown to increase activity of PGAP, Singleton M. *Thesis*, University of Exeter, 1997). 10 µl of the PGAP solution (2 mU) was added to 10 µg HL4E10 IgG in 15 µl of 50 mM Na-phosphate, 1 mM EDTA. 0.1% Tween 20 was the added to the reaction mix to improve enzymatic cleavage [36]. After the reaction was incubated for 6 h at 40°C, a second 10 µl aliquot of the reconstituted enzyme (2 mU) was added and the incubation continued at 40°C for another 4 h. After SDS-PAGE, the samples were blotted onto a PVDF membrane and the band corresponding to the HL4E10 heavy chain was submitted for Edman degradation. N-terminal sequencing (after the PGAP removal of the N-terminal Gln) yielded VQLKESGPGL.

### cDNA sequencing of the HL4E10 hamster IgG

Degenerate oligonucleotide primers for HL4E10 light and heavy chains were designed from the N-terminal sequences (light chain degenerate sense primer: 5′TAYACNYTNACNCARCCNCCNYT3′; heavy chain degenerate sense primer: 5′CARGTNCARYTNAARGARWSNGGNCCNGGNYTN3′). RNA was isolated from HL4E10 mAb-secreting hybridoma cells using Trizol (Invitrogen). cDNA was synthesized from total RNA and HL4E10 cDNA amplified by 3′RACE PCR using an RLM-RACE kit (Ambion) and the above gene-specific 5′ primers. PCR products were cloned into pCR 2.1-TOPO (Invitrogen) and subjected to DNA sequencing using M13 forward and reverse primers.

To obtain the sequence of the 5′UTR, specific antisense primers were designed based on the sequences obtained from the 3′RACE PCR products. Primers were as follows; light chain antisense: 5′ATTGGGCTGTACCTAGGACAGT3′ and heavy chain antisense: 5′TCATTTACCGGGCCTCTGGGACAGA3′. RNA was subjected to 5′RACE PCR using an RLM-RACE kit and the above gene-specific 3′ primers. PCR products were cloned and sequenced as described above. Additional gene-specific primers were used to verify sequences such that each base was verified at least twice using PCR products obtained from independent primer sets. The complete cDNA sequences of the HL4E10 light chain and HL4E10 heavy chain have been deposited in Genbank (accession numbers HM369134 and HM369133, respectively).

### Sequence analysis and comparisons

For hierarchical clustering, signal peptides were predicted using SignalP 3.0 [37], [38] and removed from their Ig light and heavy chain amino-acid sequences before alignment with MUSCLE [39]. Distance matrices, where distance is measured as the number of amino acid substitutions per site, were calculated in MEGA4 [40] using 13 heavy chain amino acid sequences and 22 light chain amino acid sequences and applying the Poisson correction model [41]. Hierarchical clustering of distances was performed with MultiDendrograms v2.1.0 [42] and displayed using the same program. Sequence identities were calculated using the Smith-Waterman algorithm [43], [44]. Identification of rare amino-acid residues at particular positions and Chothia canonical class assignments using auto-generated SDR templates [45] were performed with AbCheck [46], [47]. Analysis of variable region homology of the HL4E10 cDNA sequence to known human, mouse and rat germline V, D and J sequences was conducted using IMGT/V-QUEST [48].

### Preparation and purification of HL4E10 Fab

Hybridoma cells secreting HL4E10 hamster IgG were cultured in DMEM complete medium (Gibco, Invitrogen) with the following supplements: 20% FCS, 2 mM L-Glutamine, 25 mM HEPES buffer, 1 mM Na-pyruvate, 100 mM non-essential amino acids, 100 U penicillin, 100 µg streptomycin, and 1x vitamins (Irvine Scientific). Three liters of supernatant (added 0.02% NaN_3_) were adjusted to pH 8.0 with 300 ml 1.0 M Tris-Cl pH 8.0 and the IgG was bound to a Protein A-Sepharose (GE Healthcare, BioRad) column. After washing with 500 ml of Protein A binding buffer (0.1 M Tris-HCl, 3.0 M NaCl, 0.01 M Na-EDTA, pH 8.9), the IgG was eluted with Protein A elution buffer (0.10 M acetic acid, 0.15 M NaCl, pH 3.0) and immediately neutralized with 100 mM NaHCO_3_ pH 9.0. The IgG was digested with 4% Pepsin in 1.0 M Na-acetate pH 5.5 in the presence of 20 mM cysteine. The reaction was stopped after 3 hours by addition of 1/7th volume of 1.0 M Tris-Cl pH 10. Undigested IgG and Fc fragments were removed by exploiting their affinity for a Protein A column. The unliganded Fab was further purified to homogeneity on Protein G and Superdex 75 16/100 columns.

### Crystallization and data collection

The HL4E10 hamster IgG Fab (7 mg/ml) was crystallized from 10–12.5% PEG 4000, 0.1 M Na-acetate pH 4.6, 0.2 M (NH_4_)_2_SO_4_ at 22°C by sitting drop vapor diffusion by mixing 0.5 µl protein solution with 0.5 µl reservoir solution. Crystals nucleated overnight and grew to their final size of 0.06×0.03×0.02 mm within a week. A complete data set to 2.95 Å was collected at the Stanford Synchrotron Radiation Lightsource beamline 11–1 (Palo Alto, USA) and was integrated and scaled with HKL2000 [49].

### Structure determination, refinement, and analysis

The structure of the HL4E10 Fab was determined by molecular replacement (MR) to 2.95 Å resolution in monoclinic space group P2_1_ (V_M_ = 2.4 Å^3^/Da for two molecules per ASU). Using the FFAS03: Fold, Function and Assignment Server [50], the HL4E10 Fab light and heavy chain sequences were threaded onto the coordinates of the light and heavy chains of Fabs with the highest sequence identity to HL4E10: the HYB3 Fab light chain (1W72, 65% sequence identity) and the YTS 105.18 Fab heavy chain (2ARJ, 74% sequence identity). Using the SCWRL server {JCSG, Joint Center of Structural Genomics, La Jolla, USA (jcsg.org)}, an all atom model, which retained original rotamers for the conserved residues, was generated for the individual light and heavy chain. The chains were then reassembled into the V_L_:V_H_ and C_L_:C_H_1 regions of an Fab molecule and MR solutions were found using PHASER [51].

The MR model was subjected to rigid body refinement and restrained all atom refinement with simulated annealing using CNS [52]. Further refinement was achieved by alternating cycles of model building with COOT [53] and refinement with CNS and REFMAC5 [54]. The final model was refined to R_cryst_ = 22.7% and R_free_ = 28.1% (Table 1) and consists of two HL4E10 Fabs (Fab 1: chain L residues 1–211, chain H residues 1–228; Fab 2: chain A residues 1–211, chain B residues 1–228) per asymmetric unit. No solvent molecules were added to the model due to the moderate resolution of the structure determination. The final statistics are shown in Table 1. The quality of the structure was evaluated with PROCHECK [55], WHATCHECK [46], and MOLPROBITY [56]. Superimpositions of the Cα atoms of the entire Fab molecules LH and AB were done with SSM; superimpositions of all atoms of the individual light and heavy chains with LSQ, as implemented in COOT. Coordinates and structure factors have been deposited in the PDB Protein Data Bank with accession number 3MJ8.

**Table 1:** Hamster HL4E10 Fab data collection and refinement statistics.

	HL4E10 Fab
Data collection	
Space group	P2_1_
Cell dimensions	
*a*, *b*, *c* (Å)	43.8, 148.8, 68.8
α, β, γ (°)	90.0, 106.2, 90.0
Wavelength (Å)	0.97946
Resolution (Å)	30.00–2.95 (3.06–2.95)*
*R*_merge_ (%)	10.5 (54.1)*
*<I*/σ*I>*	7.7 (1.7)*
Completeness (%)	95.3 (96.1)*
Unique reflections	17,012
Redundancy	2.3
**Refinement**	
Resolution (Å)	30.00–2.95
No. reflections work/test	16,120/863
*R*_work_/*R*_free_ (%)	22.7/28.1
No. atoms	
Light chain L	1575
Heavy chain H	1585
Light chain A	1575
Heavy chain B	1585
*B*-values (Å^2^)	
Light chain L	53
Heavy chain H	54
Light chain A	56
Heavy chain B	56
R.m.s deviations	
Bond lengths (Å)	0.006
Bond angles (°)	0.96
Ramachandran stats	
Favored/allowed/outliers (%)	95.2/4.7/0.1#

*Highest resolution shell is shown in parenthesis.

# Residue Asp^L170^ is the only residue in the disallowed region, but is located in region closely resembling a γ-turn.

### Structure prediction and modeling

Structure prediction and generation of automated models of HL4E10 was done with PIGS [57]. First, the three independent heavy chains and light chains that best matched the canonical structures of HL4E10 were identified (HC: 1W72, 2G75, 1ADQ; LC: 1A7P, 1GIG, 1DL7). Next, while keeping loops with similar canonical structures (n.b. HL4E10 CDRL1 and CDRL3 did not match to any canonical structures of the templates and remained not defined), side chains were modeled by transferring conserved residues and predicting the conformations of non-conserved side chains with SCWRL3.0 [58] for each of the three HC and three LC templates. The resulting HC and LC variable domain models were then superimposed with the experimentally determined HL4E10 structure using SSM [59].

## Results and Discussion

### A mild protocol to remove N-terminal pyroglutamate from reduction-sensitive proteins

To yield N-terminal protein sequences for primer design and determination of the cDNA sequence of the stimulatory hamster antibody HL4E10, we developed an optimized protocol for enzymatic removal of N-terminal pyroglutamate residues from reduction-sensitive proteins. Glutamine cyclization to pyroglutamate is frequently found in proteins (n.b. most immunoglobulins contain glutamine at the amino terminus of their heavy chains) and inhibits N-terminal sequencing by Edman degradation [34], [60]. Pyroglutamates can be enzymatically removed using pyroglutamate aminopeptidase (PGAP); however, the target protein has to be stable under reducing conditions to prevent oxidation of the PGAP catalytic cysteine [35]. Therefore, cleavage with PGAP is usually conducted in the presence of 10 mM DTT, which, as for HL4E10 IgG, can affect the integrity of proteins with disulfide bonds [34]. We screened different concentrations of reducing agents, and reaction temperatures and found that, after an initial 2 min heat activation of PGAP at 55°C, 1 mM β-mercaptoethanol in the reaction mix is sufficient for PGAP activity. The HL4E10 IgG did not degrade under these conditions and the pyroglutamate at the N-terminus of the heavy chain was successfully removed during 10 h incubation at 40°C. Our modified, milder protocol for enzymatic removal of pyroglutamate residues using PGAP should be generally useful for N-terminal sequencing of reduction-sensitive proteins with pyroglutamate-blocked N-termini.

### The hamster HL4E10 IgG sequence in comparison to other IgGs

**Figure 1:**
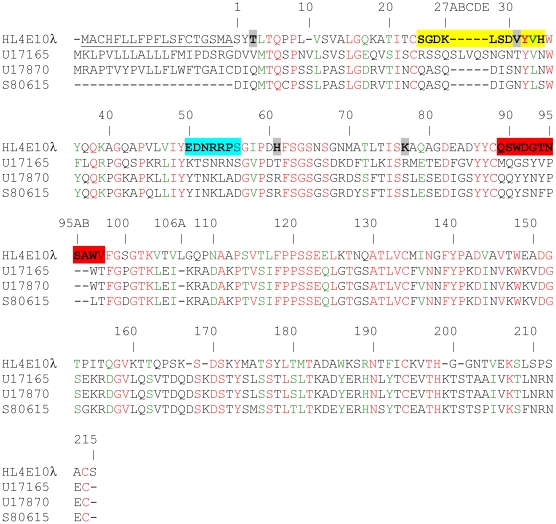
Amino-acid sequence alignment of the HL4E10 hamster IgG light chain with those of other hamster antibodies. The HL4E10 lambda light chain is aligned with H28.710 (Genbank accession no. U17165 [32]), 145.2c11 (U17870 [31]), and 1F4 (S80615 [30]). Identical residues are in red and homologous exchanges are in green. Signal peptides are underlined, CDR loops are shaded: CDR L1 is in yellow, CDR L2 is in cyan, CDR L3 is in orange-red, and residues which are rarely observed in antibodies at particular locations are shaded gray. Kabat numbering is used throughout, as well as the definition of CDRs.

**Figure 2:**
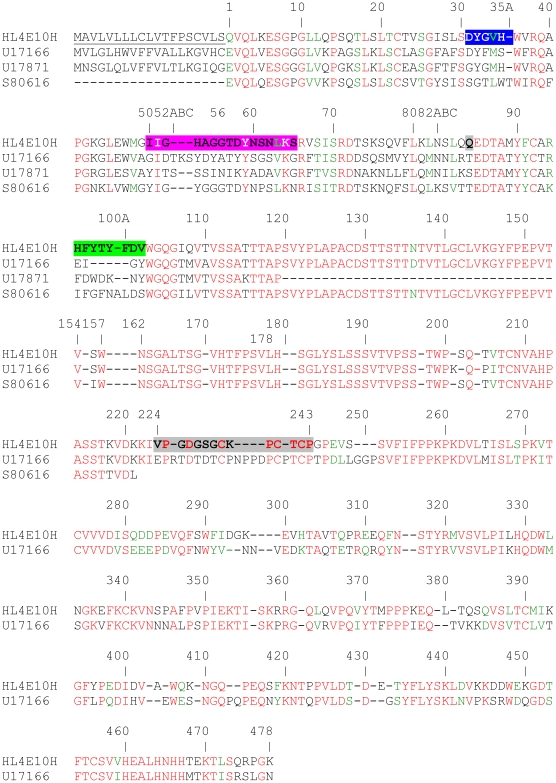
Amino-acid sequence alignment of the HL4E10 hamster IgG heavy chain with those of other hamster antibodies. The HL4E10 heavy chain is aligned with H28.710 (U17166 [33]), 145.2c11 (U17871 [31]), and 3A5-1 (S80616 [30]). Color coding and shading is used as in Fig. 1, CDR H1 is in blue, CDR H2 in pink, CDR H3 in green, and the glycine-, proline-, cysteine-rich hinge region between V_H_C_H_1 and C_H_2C_H_3 is shaded gray.

Using degenerate primers derived from N-terminal protein sequences, we determined the complete cDNA sequence for the hamster IgG HL4E10. The light chain cDNA comprises 699 base pairs (Genbank accession no. HM369134) encoding a 19-residue signal peptide and a 213 amino acid mature protein chain (Fig. 1). The HL4E10 heavy chain (HM369133) comprises 1389 base pairs encoding a 19-residue signal peptide and a 443-residue mature protein chain (Fig. 2).

**Figure 3:**
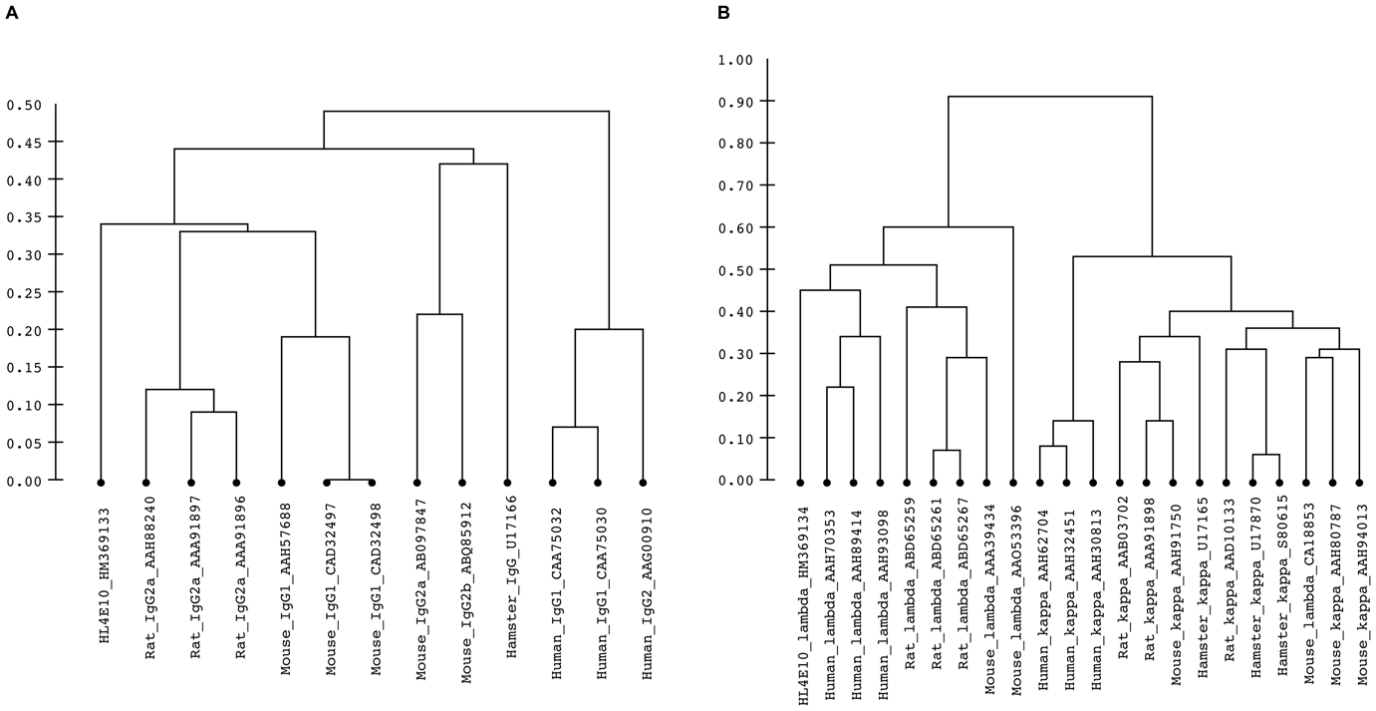
Hierarchical clustering of the HL4E10 protein sequence with known immunoglobulins. Distances were calculated between protein sequences of (**A**) heavy chains and (**B**) light chains as the number of amino acid substitutions per site. Scale bar indicates distances. GenBank accession numbers of sequences included in the analysis are indicated within branch labels. Hierarchical clustering was performed on distance matrices generated from protein sequences with removed signal peptides.

While phylogenetic analysis of HL4E10 relative to other antibodies was precluded by the unavailability of its germline sequence, the mature sequence was analyzed to infer its overall homology to known rearranged antibodies and identify the most likely germline V(D)J constituents. According to pairwise distance measurements between immunoglobulin sequences, calculated as the number of amino acid substitutions per site, the HL4E10 heavy chain is generally more homologous to rodent IgG heavy chains than to human IgG heavy chains (Fig. 3A). Average distance from HL4E10 heavy chain sequence to the rat subgroup IIa sequences shown in the dendrogram is 0.35 and 0.36 to the mouse IgG1 subgroup sequences, while average distances to the human IgG1 and IgG2 clades are 0.45 and 0.51, respectively. Mouse subgroup IIa/b seems to differ from both its rat and human homologs, which is probably why it appears to be less similar to the HL4E10 sequence than other rodent sequences. The only other known hamster heavy chain sequence contains 0.39 substitutions per site relative to HL4E10 heavy chain. To put distance measurements into a more intuitive perspective, HL4E10 shares 73.2% sequence identity with the rat IgG2a heavy chain (GenBank ID AAH88240), 73.4% with the mouse IgG1 (AAH57688), 61.3% with the human IgG2 (CAA75032) and 65.4% with the other known hamster heavy chain sequence (U17166). When compared to germline VDJ regions of other species, HL4E10 cDNA sequence shares the most homology with rat IGHV2 region (89% identity), mouse IGHD5 region (100%) and human IGHJ4 region (83%).

Interestingly, the HL4E10 light chain is marginally more homologous to human lambda light chains than to rodent lambda light chains (Fig. 3B). The average distance to human lambda is 0.42, while rat lambda chains have on average 0.58 and mouse 0.64 substitutions per site, respectively. Correspondingly, HL4E10 shares 64.5% sequence identity with a human lambda sequence (GenBank ID AAH70353), 59.8% with a rat (ABD65259) and 58.4% with a mouse lambda sequence (AAA39434). Alignment to germline human, rat, and mouse sequences showed that HL4E10 light chain is most homologous to the human IGLV3 region and human IGLJ3 region. The HL4E10 lambda light chain sequence is dissimilar to the three known hamster kappa light chain IgG sequences (0.82 substitutions per site and only 40.3%, 40.7% and 40.3% sequence identity with the framework residues of U17165, U17870, and S80615, respectively), whereas three hamster kappa light chain sequences show only 0.24 amino acid substitutions per site on average and 79.1% framework residue sequence identity with each other. Rat, mouse and human kappa sequences are also dissimilar from the HL4E10 sequence, further supporting the classification of the HL4E10 light chain as of the lambda type.

The HL4E10 variable sequence exhibits five residues which are rarely observed at certain positions in antibodies: Thr^L3^ is present in only 0.70% of antibodies, Val^L31^ (0.80%), His^L61^ (0.06%), Lys^L77^ (0.48%), and Gln^H84^, which is located in FR3, has never been observed before at that location in an antibody sequence. An interesting feature of the HL4E10 heavy chain sequence is the glycine-rich hinge region connecting C_H_1 and C_H_2 (Fig. 2), which might contribute to the ready degradation of the HL4E10 heavy chain into the V_H_C_H_1 and C_H_2C_H_3 fragments in the presence of reducing agents without any added proteases (as discussed above).

The HL4E10 CDR sequences can be assigned to the following Chothia canonical classes: CDR H1, class 1/10A; CDR H2, class 1/9A; CDR L1, similar to class 2/11A, but differs in residues Tyr^2^ (Chothia allowed: Ile), Gly^25^ (Ala), Asp^26^ (Ser), Leu^28^ (Asn, Ser, Asp, Glu), Ser^29^ (Ile, Val), Asp^51^ (Ala, Thr, Gly, Val), Ala^71^ (Tyr, Phe), Ser^90^ (His, Gln); CDR L2, class 1/7A; CDR L3, similar to class 5/11A but with Gln^89^ (instead of Chothia allowed: Ala) and Ser^90^ (Ala). Furthermore, the CDR H3 sequence features a high percentage (50%) of aromatic residues with two tyrosines and two phenylalanines out of 8 residues (Fig. 2).

### The crystal structure of the HL4E10 Fab

**Figure 4:**
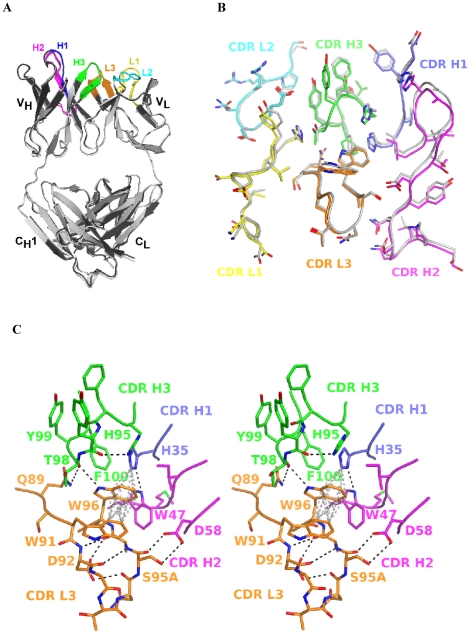
Crystal structure of the HL4E10 Fab. (**A**) Cartoon representation of the superimposition of the two HL4E10 Fab structures in the asymmetric unit. The two HL4E10 Fabs (LH and AB) are shown in dark and light gray, respectively. The CDR loops are color coded as in Fig. 1&2: CDR L1 yellow, CDR L2 cyan, CDR L3 orange, CDR H1 blue, CDR H2 pink, CDR H3 green. The Cα atoms of Fab LH and Fab AB superimpose with an r.m.s.d. of 0.63 Å. (**B**) Superimposition of the combining sites of HL4E10 Fab LH (CDR loops colored) and Fab AB (CDR loops gray) (in a similar orientation to Fig. 4C) reveals a rigid assembly without significant conformational differences. (**C**) Wall-eyed stereo representation of the molecular interactions which rigidify the HL4E10 CDR loops and lock the side chains in conformations predefined for high affinity ligand binding. For example, hydrogen bonds (black), CH-π interactions (grey), and hydrophobic stacking interactions occur at the interface of CDR L3 with CDRs H3, H2, and H1.

The crystal structure of the hamster HL4E10 Fab resembles that of a typical Fab [61]. The two HL4E10 Fab molecules (chains LH and AB) in the asymmetric unit do not significantly differ from each other in their overall structure or in the conformation of the combining site (Fig. 4A,B). The Cα atoms of the two Fabs in the asymmetric unit superimpose with an r.m.s.d. of 0.63 Å; all atoms of the light chains L and A superimpose with an r.m.s.d. of 0.79 Å; and all atoms of the heavy chains H and B with an r.m.s.d. of 0.63 Å. The elbow angles between the variable and constant domains are 193.5° and 189.5° for chains LH and AB, respectively. Because of the structural similarity of the two Fabs in the asymmetric unit, we will limit our discussion to the structural properties of Fab LH.

**Figure 5:**
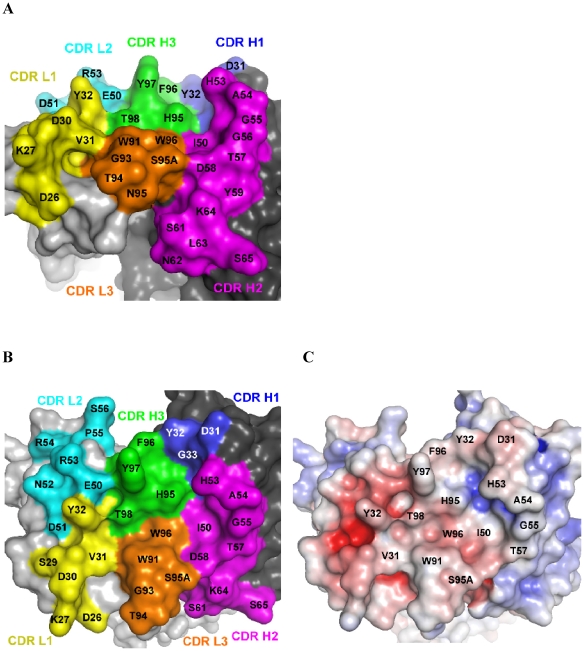
Molecular surface representations of the HL4E10 combining site. (**A**) Side view of the combining site in a similar orientation to Fig. 4B. The central region is formed by Val^L31^, Trp^L96^, Ile^H50^, His^H95^, Phe^H96^, and Thr^H98^ and surrounded by Asp^H31^ and Tyr^H32^ of CDR H1; His^H53^-Asp^H58^ of CDR H2; Asp^L30^ and Tyr^L32^ of CDR L1; Arg^L53^ of CDR L2; and Trp^L91^ and Ser^L95A^ of CDR L3. Tyr^L32^ and Tyr^H97^ rise significantly above the central plain of the combining site and Tyr^L32^, Glu^L50^, Tyr^H97^, and Thr^H98^ form a pocket on the HL4E10 surface. (**B**) View of the ligand-binding site after a 60° rotation of (A) around the horizontal axis. (**C**) The electrostatic potential was mapped onto the molecular surface in the same orientation as (B) and contoured at ±10 kT/eV (blue/red). The largely hydrophobic character (white) of the HL4E10 combining site is defined by six aromatic (Tyr^L32^, Tyr^H32^, Tyr^H97^, Phe^H96^, Trp^L91^, and Trp^L96^) and two aliphatic (Val^L31^ and Ile^H50^) residues.

The HL4E10 combining site is composed of a relatively flat central area (formed by Val^L31^, Thr^H98^, Trp^L96^, Ile^H50^, His^H95^, and Phe^H96^), surrounded by more protruding residues (Asp^H31^, Tyr^H32^ of CDR H1; His^H53^-Asp^H58^ of CDR H2; Asp^L30^, Tyr^L32^ of CDR L1; Arg^L53^ of CDR L2; and Trp^L91^ and Ser^L95A^ of CDR L3) (Fig. 5A,B). A number of aromatic residues, i.e. three tyrosines (Tyr^L32^, Tyr^H32^, Tyr^H97^), one phenylalanine (Phe^H96^), two tryptophans (Trp^L91^, Trp^L96^), and two histidines (His^H35^, His^H95^); and two aliphatic residues (Val^L31^, Ile^H50^) render the center of the HL4E10 combining site rather hydrophobic in nature (Fig. 5C).

The side chains of two residues, namely Tyr^L32^ and Tyr^H97^, rise significantly above the central plain of the combining site (Fig. 5A), but do not exhibit any significant differences when comparing both HL4E10 molecules in the asymmetric unit (Fig. 4B), suggesting that the HL4E10 combining site is mostly rigid and structurally optimized for ligand binding. The rigid arrangement of the HL4E10 CDR loops is maintained by an extensive network of hydrophobic stacking and CH-π interactions [62], [63], and is further stabilized by H-bonds between different CDR loops, as well as between CDR loops and framework residues (Fig 4C). For example, in the CDR L3 loop, Trp^L91^ engages in a CH-π interaction with Trp^L96^, which H-bonds with Thr^H98^, and is also involved in a CH-π interaction with His^H35^. The latter residue stacks against Phe^H100^ and H-bonds with Trp^H47^. In the CDR H3, 5 out of 6 consecutive residues have large, aromatic side chains (His^H95^, Phe^H96^, Tyr^H97^, Tyr^H99^, Phe^H100^) and are tightly packed between the CDR H1, L1, L2, and L3 loops, thus constricting any significant motion (Fig. 4B). Taken together, the rather rigid HL4E10 combining site serves as a structural framework to optimally position the CDR residues for HL4E10-JAML interaction [27] prior to antigen binding.

**Figure 6:**
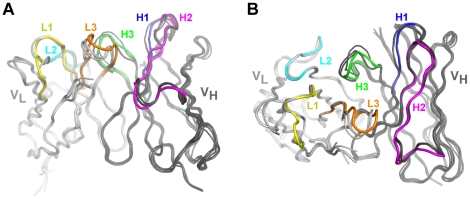
Structure prediction and automated modeling of HL4E10. Cartoon representation of the superimposition of the experimentally determined HL4E10 heavy and light chain variable domain structures and the three top scoring heavy (1W72, 2G75, 1ADQ) and light chain (1A7P, 1GIG, and 1DL7) computational models. Heavy and light chains are shown in dark and light gray, respectively. The CDR loops of HL4E10 are color coded as in Fig. 1&2: CDR L1 yellow, CDR L2 cyan, CDR L3 orange, CDR H1 blue, CDR H2 pink, CDR H3 green. The Cα atoms of the experimentally determined HL4E10 structure and the computational models superimpose well, with an average r.m.s.d. of 0.68 Å for the heavy chains and 0.77 Å for the light chains, respectively. The largest deviations are observed, as expected, in the CDR loops, namely L1, L3 and H3.

As HL4E10 is the first example of a three-dimensional structure of a hamster antibody, its experimental structure was compared to computationally predicted structures generated from canonical structure search (Fig. 6). The three top scoring computational heavy chain variable domain models, 1W72, 2G75, and 1ADQ, superimpose with HL4E10 with r.m.s.d.'s of 0.67, 0.60, and 0.78 Å, respectively. For the LCs, the three top scoring variable domain models 1A7P, 1GIG, and 1DL7 superimpose with HL4E10 with RMSDs of 0.83, 0.66, and 0.81 Å, respectively. As expected, the largest deviations are seen for CDR H3 (r.m.s.d. 's of 1.0, 0.77, 0.99 Å), as well as for CDR L1 (r.m.s.d. 's of 0.80, 0.73, 0.81 Å) and CDR L3 (r.m.s.d. 's of 0.90, 0.94, 1.20 Å) whose canonical classes could not be unambiguously assigned. Nevertheless, given that no structural information has been previously available for hamster IgGs, the computationally predicted structure models compare surprisingly very well to the experimentally determined HL4E10 structure.

### Conclusion

We have determined the complete cDNA sequence and the three-dimensional structure of the Fab fragment of a hamster antibody stimulatory for γδ T cells. The primary structure of HL4E10 is the first example of a hamster lambda light chain sequence, and its heavy chain sequence is only the second known complete hamster heavy chain sequence. Thus, the cDNA sequence of HL4E10 significantly extends our limited knowledge of hamster immunoglobulin sequences. The crystal structure of the unliganded HL4E10 Fab reveals an essentially rigid combining site which is already conformationally optimized for interaction with its ligand JAML. The binding of HL4E10 to γδ T cell-expressed JAML induces potent costimulation and ultimately cell proliferation, suggesting that humanized HL4E10 derivatives, might be useful therapeutic tools for treatment of γδ T cell dysfunction-associated diseases, such as chronic non-healing wounds or cancer.
